# Supplementary material for: Segregated Dynamical Networks for Biological Motion Perception in the Mu and Beta Range Underlie Social Deficits in Autism
Source: Diagnostics (Basel). 2024 Feb 13;14(4):408. doi: 10.3390/diagnostics14040408 (PMC10887711; doi:10.3390/diagnostics14040408)
Supplement: Supplementary file 1 [file diagnostics-14-00408-s001.zip › Supplementary_Table S1.pdf]

No. Of. Connections	Mu-TDC	ED	Beta-TDC	ED
1	1-2	84.083	1-2	95.796
2	1-3	60.737	2-1	95.796
3	3-1	60.737	1-3	94.037
4	5-1	110.806	5-1	106.475
5	2-3	67.742	5-2	76.459
6	2-4	93.669	3-4	74.263
7	4-2	93.669	4-3	74.263
8	5-3	72.076	3-5	70.338
9	4-5	48.166	5-3	70.338
10	5-4	48.166		
		73.985 (Mean)		84.196 (Mean)

No. Of. Connections	Mu-ASD	ED	Beta-ASD	ED
1	1-2	62.498	1-3	76.980
2	1-3	67.512	2-3	74.525
3	3-1	67.512	3-2	74.525
4	2-3	40.274	3-4	80.566
5			4-3	80.566
		59.449 (Mean)		76.649 (Mean)
