# Supplementary figures and images for: Segregated Dynamical Networks for Biological Motion Perception in the Mu and Beta Range Underlie Social Deficits in Autism
Source: Diagnostics (Basel). 2024 Feb 13;14(4):408. doi: 10.3390/diagnostics14040408 (PMC10887711; doi:10.3390/diagnostics14040408)

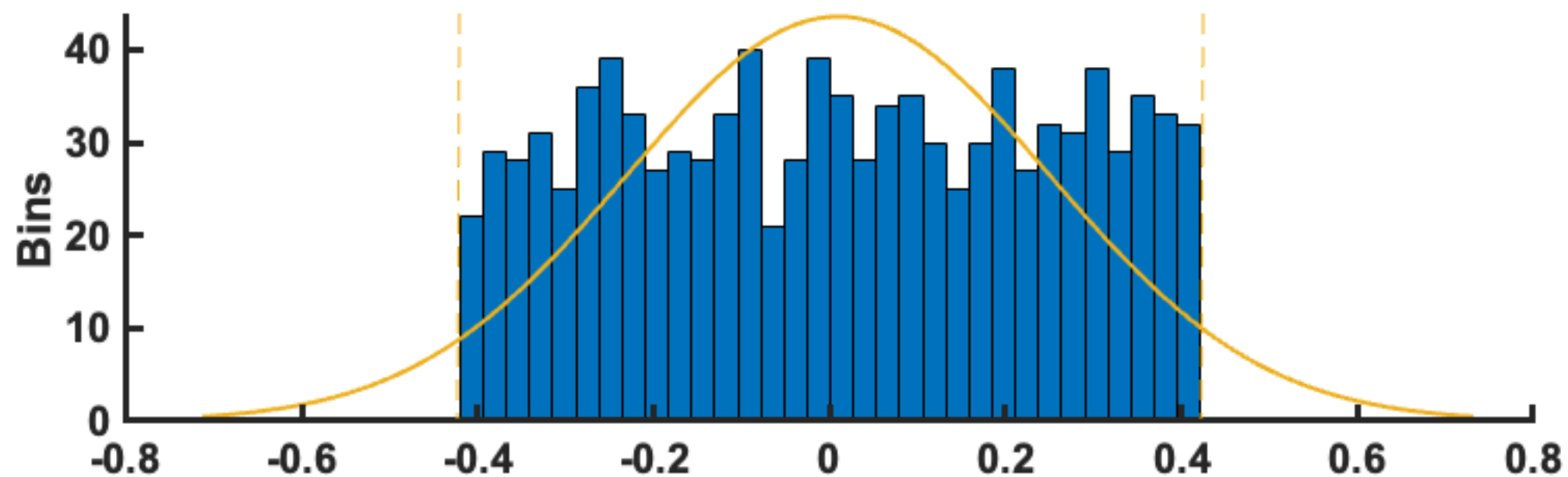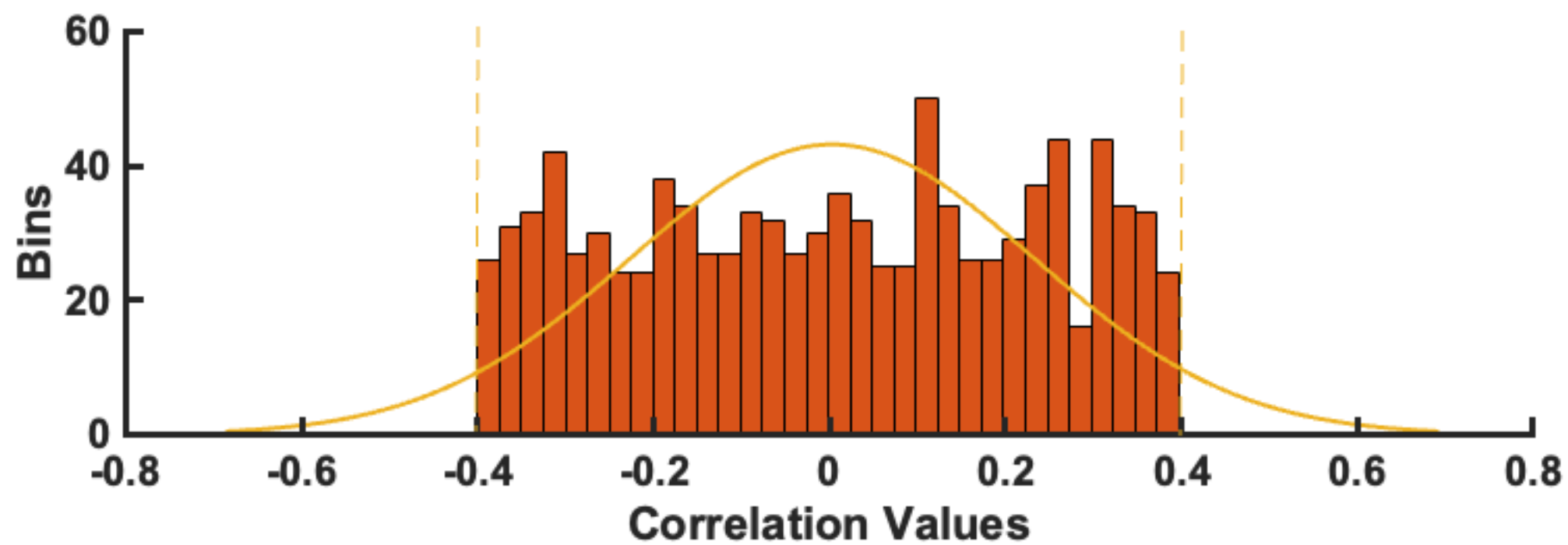

Supplement: Supplementary file 1 [file diagnostics-14-00408-s001.zip › Suppl_FigureS1.pdf]
