# Supplementary material for: Segregated Dynamical Networks for Biological Motion Perception in the Mu and Beta Range Underlie Social Deficits in Autism
Source: Diagnostics (Basel). 2024 Feb 13;14(4):408. doi: 10.3390/diagnostics14040408 (PMC10887711; doi:10.3390/diagnostics14040408)
Supplement: Supplementary file 1 [file Suppl_FigureS2.pdf › Suppl_FigureS2.pdf]

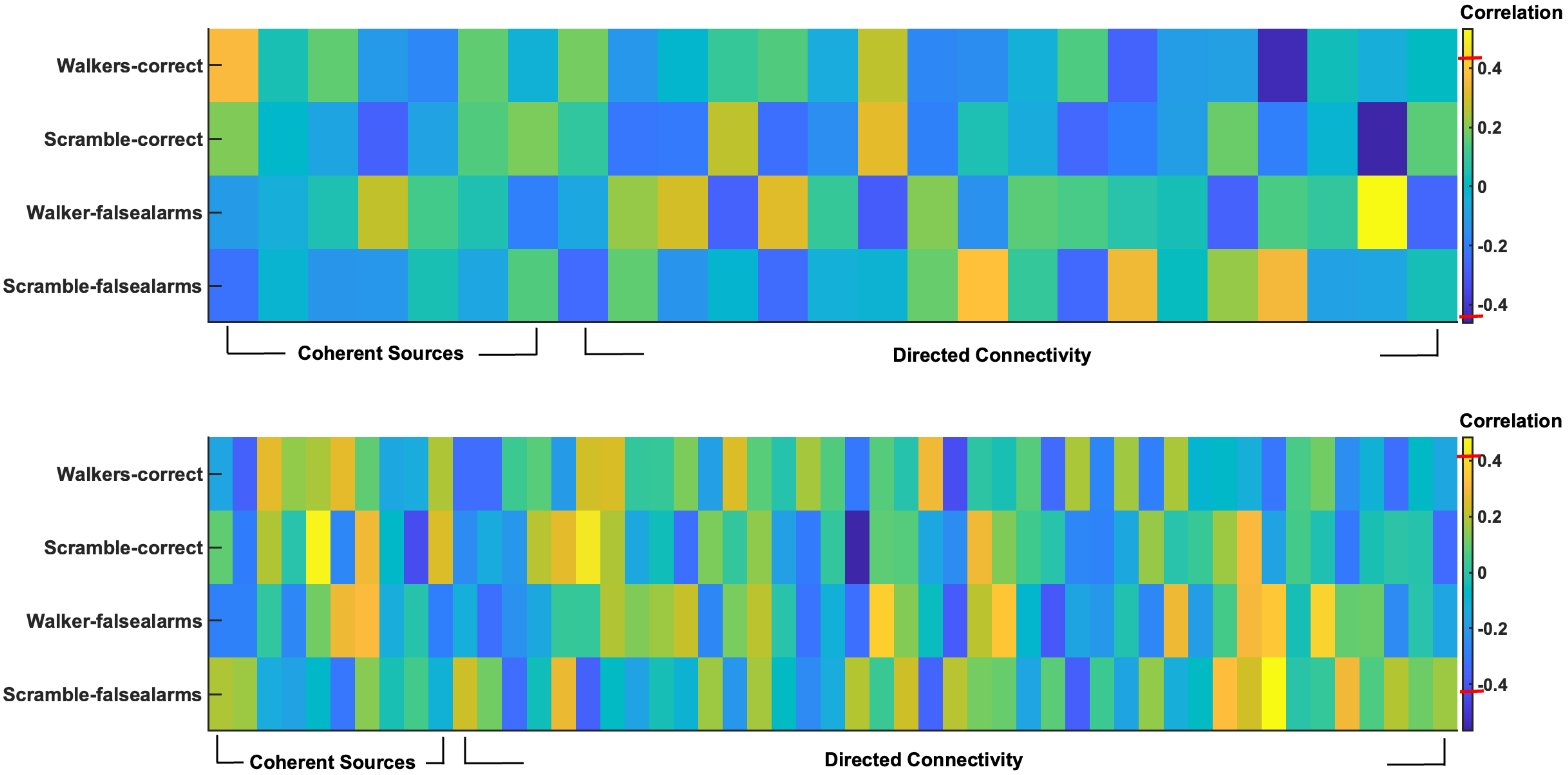
Correlation
0.4
0.2
0
-0.2
-0.4
Walkers-correct
Scramble-correct
Walker-falsealarms
Scramble-falsealarms
Coherent Sources
Directed Connectivity
Correlation
0.4
0.2
0
-0.2
-0.4
Walkers-correct
Scramble-correct
Walker-falsealarms
Scramble-falsealarms
Coherent Sources
Directed Connectivity
